# Chemerin/CMKLR1 Axis Promotes the Progression of Proliferative Diabetic Retinopathy

**DOI:** 10.1155/2021/4468625

**Published:** 2021-11-24

**Authors:** Lihui Wang, Ying Zhang, Yanan Guo, Wencui Ding, Ailing Chang, Jing Wei, Xinsheng Li, Hongxia Qian, Chonggui Zhu

**Affiliations:** ^1^Department I of Endocrinology and Diabetes Mellitus, Cangzhou Central Hospital, Cangzhou 061000, Hebei, China; ^2^Department of Endocrinology and Metabolism, Tianjin Medical University General Hospital, No. 154 Anshan Road, Heping District, Tianjin 300052, China; ^3^Department IV of Medicine, Tangshan Likang Hospital, Xinglong Street, Han Town, Lubei District, Tangshan 063000, Hebei, China

## Abstract

**Background:**

Diabetic retinopathy (DR) is a prevalent microvascular complication of diabetes, and the levels of chemerin were associated with the severity of DR. However, there is no research on chemerin in the development of proliferative diabetic retinopathy (PDR). Therefore, our study aimed to explore the relationship between chemerin and PDR.

**Methods:**

The levels of chemerin/chemokine-like receptor (CMKLR1), proinflammatory cytokines, and vascular endothelial growth factor (VEGF) in 90 cases of PDR and nonproliferative diabetic retinopathy (NPDR) patients and in high glucose (HG) stimulated human retinal pigment epithelium cells (ARPE-19) were evaluated by ELISA. Moreover, chemerin was added into HG-induced ARPE-19 cells to assess its effect on proinflammatory cytokines and VEGF.

**Results:**

The levels of chemerin/CMKLR1 were higher in PDR patients than NPDR ones, and chemerin was positively correlated with CMKLR1 in PDR patients. Compared to NPDR, the secretions of proinflammatory cytokines and VEGF were increased in PDR patients and positively correlated with chemerin/CMKLR1. Additionally, chemerin activated CMKLR1 and aggravated HG-induced cell injury, inflammatory responses, and VEGF expressions in ARPE-19 cells.

**Conclusion:**

Our study demonstrated that chemerin/CMKLR1 axis aggravated the progression of PDR, which suggested that inhibition of chemerin might serve as a new therapeutic approach to treat PDR.

## 1. Introduction

Diabetic retinopathy (DR) is a common diabetic microvascular complication. Its morbidity takes up a large proportion in a variety of diabetic microvascular diseases, and it can eventually cause blindness in diabetic patients. DR can be divided into two types: proliferative and nonproliferative one based on the occurrence of angiogenesis. The pathogenesis of DR remains largely unknown without systematic conclusion. However, a body of studies demonstrated that the onset of DR was associated with blood glucose control and diabetic progression in diabetic patients. Moreover, DR is also related with proinflammatory factors and lipid metabolism disorders. Long-term hypoxia in the retina upregulates the expressions of related factors, leading to the overproduction of many factors, including vascular endothelial growth factor (VEGF), stimulating the division of endothelial cells, and eventually causing a mild inflammatory response as well as proliferation in the blood vessels of retina. During this process, a variety of cytokines will be produced to stimulate neovascularization. These new blood vessels are fragile and are prone to fibrosis, hemorrhage, and rupture, which may affect the retina, resulting in a severe loss of vision.

Nowadays, current studies discovered that the expression of interleukin-1*β* (IL-1*β*) in the serum and vitreous of patients with proliferative diabetic retinopathy (PDR) was significantly higher than that of healthy people. Chemokine-like receptor 1 (CMKLR1) is an endogenous ligand of chemerin. When CMKLR1 binds to chemerin, calcium ions are released to regulate nuclear transcription factors, extracellular signal-regulated kinases-1 (ERK-1), and other signaling pathways, which play an important role in the development of cardiovascular diseases, metabolic diseases, and inflammation. CMKLR1 mainly exists in vascular smooth muscle cells, epithelial cells, endothelial cells, osteoclasts, leukocytes, and adipocytes. It interacts with IL-1*β* to activate the inflammatory cascades. Based on these observations, in our study, we explored the role of chemerin/CMKLR1 in the development of PDR.

## 2. Materials and Methods

### 2.1. Patients' Samples

The study was approved by the Ethic Committee of Cangzhou Central Hospital. 90 patients with DR were recruited and were divided into experimental group I (nonproliferative diabetic retinopathy, NPDR) and experimental group II (PDR) according to the severity of retinopathy (*N* = 45 cases for each group). Written consent was derived from the participants.

Selected patients with ages between 40 and 72 were all type II diabetes mellitus (T2DM) and agreed to our study. The patients with severe liver or kidney dysfunction, inflammatory diseases, innate immune system deficiency, or eye diseases, such as cataracts and glaucoma were excluded. Women during pregnancy and nursing or patients who used immunosuppressive agents within the past 2 months were also excluded. There was no statistically significant difference in general information between the two groups (*p* > 0.05). The sample size in this study was determined using established statistical power analysis (probability that it will reject a false null hypothesis). Differences between means of NPDR and PDR were divided by the standard deviation to determine the standardized effect size; then, using 5% as a significant difference in Student *t*-test and 90% power, the minimum required sample size was calculated.

5 ml fasting elbow vein blood samples from patients were collected and analyzed using an automatic biochemical analyzer to detect fasting plasma glucose (FPG), hemoglobin A1c (Hba1c), high-density lipoprotein cholesterol (HDL-C), total cholesterol (TC), and triglycerides (TG). LDL-C (mg/dL) = total cholesterol–HDL-C—(triglycerides/5).

### 2.2. ELISA

Blood samples from patients were centrifuged at 1500 rpm for 15 mins to acquire serum. The levels of CMKLR1, chemerin, IL-1*β*, tumor necrosis factor-*α* (TNF-*α*), and VEGF were determined using commercially available ELISA kits, respectively (Abcam, USA), according to the manufactures' instructions.

### 2.3. Cell Culture

Human retinal pigment epithelium cell line (ARPE-19) was purchased from the Shanghai Institute for Biological Sciences of the Chinese Academy of Sciences. Cells were cultured in Dulbecco's Modified Eagle Medium (DMEM) containing 10% fetal bovine serum (FBS, HyClone, Logan, USA), 1% PS (100 units/ml penicillin and 100 mg/ml streptomycin) and GlutaMAX (Gibco, USA), incubated in an incubator with 5% CO_2_ at 37°C and passaged every 3 days. When reaching approximately 80% confluence, the cells were seeded in a 6-well plate at 1.5 × 10^4^ cells and treated with different doses of D-glucose (0, 5, 10, 20, 30, 50, 75, 100 mM) for 24 h (Sigma, USA).

After starving for 24 hours, cells were stimulated with 30 mM D-glucose and different doses of recombinant human chemerin (R&D System, USA) for another 24 hours.

### 2.4. Cell Viability Assay

A colorimetric 3-(4,5-dimethylthiazol-2-yl)-2,5-diphenyltetrazolium bromide (MTT) assay was used to assess the cell viability [1]. Cells were seeded into a 96-well plate and then treated as mentioned above. MTT solution (Sigma-Aldrich, USA) was added into each well and incubated for 2-3 hours. Then, 150 ml of solution was removed from each well followed by the supplement of 100 ml isopropanol with 0.04 M HCl. After mixing thoroughly, plates were placed on the shaker at room temperature for 1 h. The absorbance was determined at 590 nm by a plate reader (BMG LABTECH, Germany). The cell viability was analyzed as the ratio of the average optical density of treated cells over control cells. IC50 was calculated from log concentration curves using nonlinear regression analysis in GraphPad Prism.

### 2.5. Western Blot

Cells were lysed in radioimmunoprecipitation buffer containing cocktail inhibitors, and then supernatant was collected after centrifugation at 12,000 g for 15 mins. Protein concentration was determined by bicinchoninic acid kit, and the same amounts of protein were loaded into sodium dodecyl sulfate-polyacrylamide gel electrophoresis gels. Western blot was performed as previously described [2].

### 2.6. RT-PCR

Total RNA was extracted from ARPE-19 cells and transcripted into cDNA. RT-PCR was performed as previously described [3]. *β-Actin* was used as control.

The sequences of the PCR primers:

 
*CMKLR*1 F: 5′-GCCAACCTGCATGGGAAAATA-3′ and R: 5′-GTGAGGTAGCAAGCTGTGATG-3′
 
*TNF-α*: F: 5′-AGCCCCCAGTCTGTATCCTT-3′ and R: 5′-CTCCCTTTGCAGAACTCAGG-3′
 
*IL-*6: F: 5′-GCCCAAACACCAAGTCAAGT-3′ and R: 5′-TATAGGAAACAGCGGGTTGG-3′
 
*IL-*1*β*: F: 5′-CAGAAGTACCTGAGCTCGCC-3′ and R: 5′-AGATTCGTAGCTGGATGCCG-3′
 
*β-Actin*: F: 5′-CGTGCGTGACATCAAAGAGAAG-3′ and R: 5′-CCAAGAAGGAAGGCTGGAAAA-3′
 
*VEG*F: F: 5′-AGGGCAGAATCATCACGAAGT-3′ and R: 5′-AGGGTCTCGATTGGATGGCA-3′

### 2.7. Statistical Analysis

Data were represented as means ± standard deviation (SD). Experiments were repeated independently for at least three times. *p* values for each group were derived from unpaired *t*' test. Chi-square test was used for assessing distribution of observations between two groups. One-way ANOVA followed by Dunnett's T3 multiple comparisons test for multiple groups. *p* < 0.05 was regarded as significant difference.

## 3. Results

### 3.1. Chemerin Was Positively Correlated with CMKLR1 in PDR Patients

We first analyzed the general information of patients, and there was no significant difference (*p* > 0.05) on gender, age, body mass index (BMI), HbA1c, HDL-C, and TC between NPDR and PDR patients (Table 1). Not surprisingly, PDR patients underwent longer duration of diabetes than NPDR patients, with higher fasting blood glucose, low-density lipoprotein cholesterol (LDL-C), and TG.

**Figure 1:**
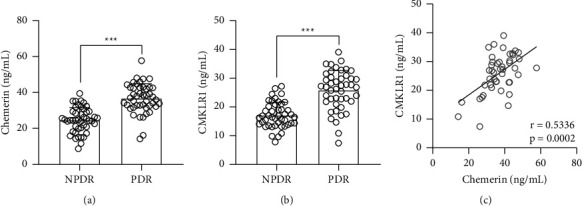
Comparisons of serum chemerin (a) and CMKLR1 (b) between NPDR and PDR patients. *N* = 45 for each group. Unpaired *t*-test. (c). Pearson correlation coefficient of serum chemerin and CMKLR1 in PDR patients.

To explore the role of chemerin in the development of PDR, we evaluated the levels of chemerin in the patients with PDR. PDR patients displayed increased expressions of chemerin and CMKLR1 in comparison with NPDR patients (Figures 1(a) and 1(b)). Furthermore, Pearson correlation showed that chemerin was positively correlated with CMKLR1 in the serum of PDR patients (Figure 1(c)).

### 3.2. Proinflammatory Cytokines Were Positively Correlated with Chemerin and CMKLR1 in the Serum of PDR Patients

**Figure 2:**
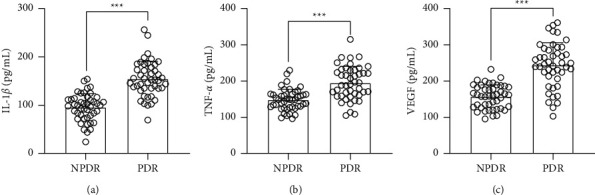
Comparisons of serum IL-1*β* (a), TNF-*α* (b), and VEGF (c) between NPDR and PDR patients. *N* = 45 for each group. Unpaired *t*-test. ^*∗∗∗*^*p* < 0.001.

**Figure 3:**
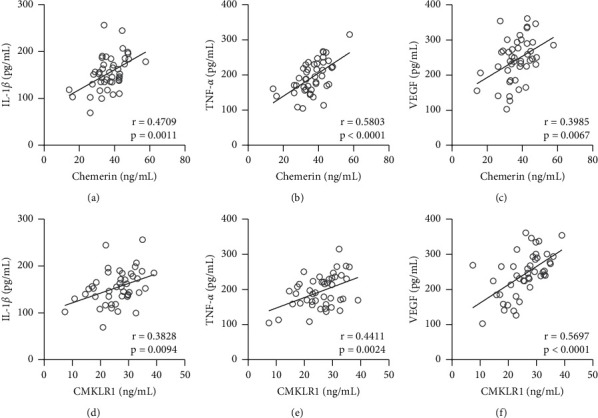
Pearson correlation coefficient of serum chemerin and IL-1*β* (a), TNF-*α* (b), and VEGF (c); serum CMKLR1 and IL-1*β* (d), TNF-*α* (e), and VEGF (f) in PDR patients.

Next, we explored the relationship between inflammation and chemerin in the PDR. Compared to that of NPDR patients, proinflammatory cytokines, including IL-1*β* and TNF-*α*, were both induced in the serum of PDR patients (Figures 2(a) and 2(b)). VEGF production was also higher in PDR patients than NPDR ones (Figure 2(c)). Furthermore, we also found that IL-1*β*, TNF-*α*, and VEGF were positively correlated with the levels of chemerin (Figures 3(a)–3(c)) and CMKLR1 (Figures 3(d)–3(f)), respectively, in the serum of PDR patients. Thus, these data suggested that either chemerin or CMKLR1 was positively correlated with the severity of inflammation in PDR patients.

### 3.3. Chemerin Activated CMKLR1 and Aggravated High Glucose-Induced Cell Injury in ARPE-19 Cells

**Figure 4:**
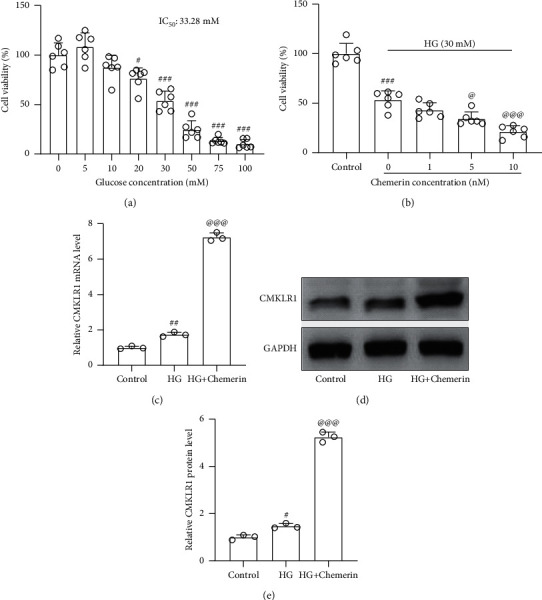
Chemerin activated CMKLR1 and aggravated high glucose-induced cell injury in ARPE-19 cells. (a) ARPE-19 cells were exposed to different concentrations of D-glucose (0–100 mM) treatment for 24 h, and then cell viability was evaluated by MTT. 30 mM was taken as high glucose (HG) concentration to stimulate ARPE-19 cells for further studies. (b) ARPE-19 cells were exposed to different concentrations of recombinant human chemerin and 30 mM HG for 24 h, and then cell viability was evaluated by MTT. 10 nM recombinant human chemerin was chosen for further experiments. *N* = 6 for each group. After 24 exposure, RT-qPCR was used to measure the mRNA levels of CMKLR1, and western blot was used to measure the protein levels of CMKLR1 (c–e). *N* = 3 for each group. ^#^*p* < 0.05, ^##^*p* < 0.01, and ^###^*p* < 0.001 compared to control, ^@^*p* < 0.05 and ^@@@^*p* < 0.001 compared to HG group. One-way ANOVA followed by Dunnett's T3 multiple comparisons test.

To further assess the role of chemerin in the PDR, we first stimulated ARPE-19 cells with different doses of D-glucose (0–100 nM) and measured high glucose (HG) induced cell injury. MTT assay identified that the IC50 of cell viability was 33.28 mM (Figure 4(a)). Therefore, we used 30 mM D-glucose for the following studies. Next, ARPE-19 cells were stimulated with 30 mM glucose and different concentrations of recombinant chemerin for 24 hours, and HG-induced cell injury was further aggravated by chemerin in a dose-dependent way (Figure 4(b)) and 10 nM chemerin was determined for the following experiments. As expected, both mRNA and protein levels of CMKLR1 induced by high glucose could be further upregulated by the stimulation of chemerin in ARPE-19 cells (Figures 4(c)–4(e)).

### 3.4. Chemerin Aggravated High Glucose-Induced Inflammatory Responses in ARPE-19 Cells

**Figure 5:**
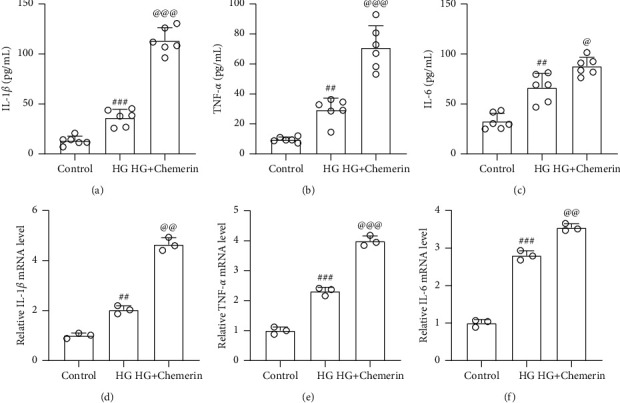
Chemerin aggravated HG-induced inflammatory responses in ARPE-19 cells. ARPE-19 cells were exposed to 10 nM recombinant human chemerin or (and) 30 mM HG for 24 h, and then ELISA was used to measure the concentrations of IL-1*β* (a), TNF-*α* (b), and IL-6 (c) in cell supernatants. *N* = 6 for each group. RT-qPCR was used to measure the mRNA levels of IL-1*β* (d), TNF-*α* (e), and IL-6 (f) from cells. *N* = 3 for each group. ^##^*p* < 0.01 and ^###^*p* < 0.001 compared to control, ^@^*p* < 0.05 and ^@@@^*p* < 0.001 compared to HG group. One-way ANOVA followed by Dunnett's T3 multiple comparisons test.

Consistent with our previous data that chemerin was correlated with inflammation in the serum of PDR patients, high glucose-induced inflammation could also be further aggravated by chemerin in ARPE-19 cells as reflected by the increased mRNA and protein levels of proinflammatory cytokines, such as IL-1*β* (Figures 5(a) and 5(d)), TNF-*α* (Figures 5(b) and 5(e)), and IL-6 (Figures 5(c) and 5(f)). These data further confirmed the relationship between chemerin and inflammatory response in HG-induced ARPE-19 cells.

**Figure 6:**
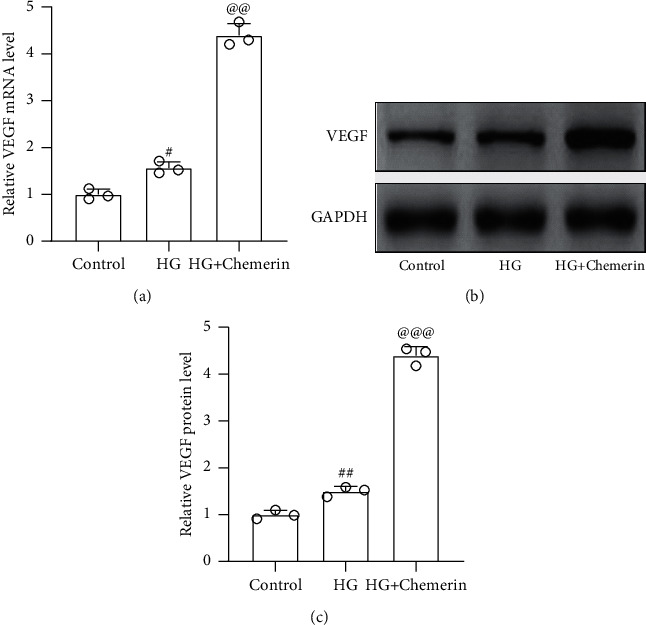
Chemerin aggravated HG-induced VEGF activation in ARPE-19 cells. ARPE-19 cells were exposed to 10 nM recombinant human chemerin or (and) 30 mM HG for 24 h RT-qPCR was used to measure the mRNA levels of VEGF (a) and western blot was used to measure the protein levels of VEGF ((b) and (c)). *N* = 3 for each group. ^#^*p* < 0.05 and ^##^*p* < 0.01 compared to control; ^@@^*p* < 0.01 and ^@@@^*p* < 0.001 compared to HG group. One-way ANOVA followed by Dunnett's T3 multiple comparisons test.

Furthermore, we also discovered that high glucose significantly increased the mRNA and protein levels of VEGF in ARPE-19 cells, which could be further upregulated by chemerin (Figures 6(a) and 6(c)). Thus, chemerin aggravated high glucose-induced VEGF activation in ARPE-19 cells, which suggested the role of chemerin/CMKLR1 in the development of PDR.

## 4. Discussion

Diabetic retinopathy, a well-recognized consequence of poorly controlled diabetes, contributes to severe vision loss and even blindness in the working age of diabetic patients. Nowadays, DR affects around 150 million people globally, and this number is estimated to double by the year 2025 according to the report of World Health Organization [4].

DR is divided into two classes: nonproliferative and proliferative, depending on whether there is neovascularization. DR without neovascularization is called NPDR, which may progress into PDR. Long-term duration of diabetes and poorly controlled hyperglycemia cause the lack of oxygen as hypoxia in the retina, which leads to the formation of new abnormal fragile blood vessels to grow along the retina [5]. Angiogenesis as well as relentless abnormal fibrovascular proliferation with bleeding and retinal detachment results in blindness [6]. Due to the importunateness of angiogenic factors, particular VEGF in the retinal angiogenesis, inhibition of VEGF is identified as a new approach to the management of PDR [7]. However, some recent studies discovered that direct anti-VEGF agents might be not as effective as expected [8]. Therefore, in our study, we explored the relationship between PDR and chemerin/CMKLR1 and found that chemerin/CMKLR1 could promote the progression of PDR by indirectly regulating the production of VEGF as well as proinflammatory cytokines.

Recent publication revealed that increased ionic calcium secretion was associated with increased ellipsoid zone disruption in DR patients with high HbA1c [9], which indicated the important role of HbA1c in DR patients. Therefore, we also analyzed the relationship between chemerin levels and HbA1c levels in the serum of patients, and the results indicated that chemerin was not correlated with HbA1c in the serum of neither NPDR nor PDR patients (*p* > 0.05, data not shown).

Chemerin is a chemoattractant protein secreted in an inactive form as prochemerin and cleavage the C-terminus to be activated. Chemerin binds to the G protein-coupled receptor CMKLR1 to activate its downstream inflammatory pathways, such as nuclear factor kappa-light-chain-enhancer of activated B cells (NF-*k*B) and ERK-1. Chemerin was discovered to stimulate chemotaxis of macrophages and dendritic cells [10]. Moreover, chemerin was identified as a novel adipokine to regulate adipocyte metabolism [11]. In addition, chemerin has been implicated to play an important role in the pathogenesis of obesity as well as diabetes [12–17]. As a chemoattractant, adipocyte-secreted chemerin was associated with insulin resistance [18]. Therefore, chemerin was also indicated to involve in revolutionizing the management of diabetic complications, such as diabetic nephropathy and DR [19]. Furthermore, the levels of chemerin were associated with the severity of diabetic retinopathy in T2DM [20–23]. However, until now, there is no publication about the relationship between chemerin and PDR. Thereby, our current study discovered that the levels of chemerin and CMKLR1 were increased in the PDR patients, and chemerin was positively correlated with CMKLR1 in the PDR patients. Our study further elucidated the role of chemerin and its receptor in the development of DR, which provided new aspects to develop the novel therapeutic approach of DR, and the small inhibitors of chemerin or CMKLR1 might serve as potential candidate drugs for DR. Since chemerin exerts its effect by binding to its receptor CMKLR1, in our study, we evaluated both the expressions of chemerin and its receptor CMKLR1 together. As a chemoattractant and adipokine, chemerin mediated the productions of proinflammatory cytokines; therefore, chemerin could be involved in the inflammation-related diseases, including diabetic retinopathy. Out study also demonstrated that the levels of chemerin/CMKLR1 were also associated with the secretions of proinflammatory cytokines, including IL-1*β*, TNF-*α*, and IL-6, in the serum of PDR patients.

## 5. Conclusion

Our current study clearly demonstrated that the levels of chemerin and its receptor CMKLR1 were higher in the serum of PDR patients than that of NPDR ones. Moreover, their productions were positively correlated with inflammatory responses and VEGF secretion in PDR patients. Therefore, the inhibition of chemerin/CMKLR1 might serve as a new therapeutic approach to manage the progression of PDR.

## Figures and Tables

**Table 1:** Demographic and clinical characteristics of the study participants.

Characteristics	Study group		*p*
	NPDR (*n* = 45)	PDR (*n* = 45)	
*Gender*			
Male	28 (62.2%)	23 (51.1%)	0.395
Female	17 (37.8%)	22 (48.9%)	
Age (years)	56.74 ± 7.11	58.19 ± 8.67	0.386
BMI (kg/m^2^)	23.65 ± 3.92	24.81 ± 3.37	0.174
Duration of DM (years)	7.8 ± 3.5	12.2 ± 4.7	**0.004**
FPG (mmol/L)	6.34 ± 1.03	7.96 ± 1.25	**0.013**
HbA1c (%)	11.29 ± 0.96	12.61 ± 1.33	0.116
HDL-C (mmol/L)	1.19 ± 0.74	1.34 ± 0.58	0.482
LDL-C (mmol/L)	2.87 ± 0.69	3.85 ± 0.52	**0.025**
TC (mmol/L)	4.39 ± 0.76	4.96 ± 0.81	0.127
TG (mmol/L)	1.79 ± 0.81	2.47 ± 0.69	**0.016**

Values were expressed as *n* (%) or mean ± SD. *p* values for each group were derived from unpaired *t* test. Chi-square test was used for assessing distribution of observations between two groups. BMI: body mass index, DM: diabetes mellitus, HbA1c: hemoglobin A1c, FPG: fasting plasma glucose, HDL-C: high-density lipoprotein cholesterol, LDL-C: low-density lipoprotein cholesterol, TC: total cholesterol, TG: triglycerides.

## Data Availability

The raw data supporting the conclusions of this article will be made available by the authors, without undue reservation.

## References

[1] Tümmler C., Snapkov I., Wickström M. (2017). Inhibition of chemerin/CMKLR1 axis in neuroblastoma cells reduces clonogenicity and cell viability in vitro and impairs tumor growth in vivo. *Oncotarget*.

[2] Liu Z., Luo H., Zhang L. (2012). Hyperhomocysteinemia exaggerates adventitial inflammation and angiotensin II−induced abdominal aortic aneurysm in mice. *Circulation Research*.

[3] Yu B., Liu Z., Fu Y. (2017). CYLD deubiquitinates nicotinamide adenine dinucleotide phosphate oxidase 4 contributing to adventitial remodeling. *Arteriosclerosis, Thrombosis, and Vascular Biology*.

[4] King H., Aubert R. E., Herman W. H. (1998). Global burden of diabetes, 1995–2025: prevalence, numerical estimates, and projections. *Diabetes Care*.

[5] Shah K. B., Han D. P. (2004). Proliferative diabetic retinopathy. *International Ophthalmology Clinics*.

[6] Crawford T., Alfaro D., Kerrison J., Jablon E. (2009). Diabetic retinopathy and angiogenesis. *Current Diabetes Reviews*.

[7] Heng L. Z., Comyn O., Peto T. (2013). Diabetic retinopathy: pathogenesis, clinical grading, management and future developments. *Diabetic Medicine*.

[8] Stewart M. W. (2012). Anti-vascular endothelial growth factor drug treatment of diabetic macular edema: the evolution continues. *Current Diabetes Reviews*.

[9] Stefanickova J., Saxena S., Nim D. K. (2019). Hyperglycemia potentiates the effect of ionic calcium in photoreceptor ellipsoid zone disruption in diabetic retinopathy. *International Ophthalmology*.

[10] Wittamer V., Franssen J.-D., Vulcano M. (2003). Specific recruitment of antigen-presenting cells by chemerin, a novel processed ligand from human inflammatory fluids. *Journal of Experimental Medicine*.

[11] Goralski K. B., McCarthy T. C., Hanniman E. A. (2007). Chemerin, a novel adipokine that regulates adipogenesis and adipocyte metabolism. *Journal of Biological Chemistry*.

[12] Bozaoglu K., Bolton K., McMillan J. (2007). Chemerin is a novel adipokine associated with obesity and metabolic syndrome. *Endocrinology*.

[13] Coimbra S., Brandão Proença J., Santos-Silva A., Neuparth M. J. (2014). Adiponectin, leptin, and chemerin in elderly patients with type 2 diabetes mellitus: a close linkage with obesity and length of the disease. *BioMed Research International*.

[14] Buechler C., Feder S., Haberl E., Aslanidis C. (2019). Chemerin isoforms and activity in obesity. *International Journal of Molecular Sciences*.

[15] Helfer G., Wu Q.-F. (2018). Chemerin: a multifaceted adipokine involved in metabolic disorders. *Journal of Endocrinology*.

[16] Zhou Z., Chen H., Ju H., Sun M. (2018). Circulating chemerin levels and gestational diabetes mellitus: a systematic review and meta-analysis. *Lipids in Health and Disease*.

[17] Tu J., Yang Y., Zhang J. (2020). Regulatory effect of chemerin and therapeutic efficacy of chemerin‑9 in pancreatogenic diabetes mellitus. *Molecular Medicine Reports*.

[18] Sell H., Laurencikiene J., Taube A. (2009). Chemerin is a novel adipocyte-derived factor inducing insulin resistance in primary human skeletal muscle cells. *Diabetes*.

[19] Shang J., Wang L., Zhang Y. (2019). Chemerin/ChemR23 axis promotes inflammation of glomerular endothelial cells in diabetic nephropathy. *Journal of Cellular and Molecular Medicine*.

[20] Yasir M., Senthilkumar G. P., Jayashree K., Ramesh Babu K., Vadivelan M., Palanivel C. (2019). Association of serum omentin-1, apelin and chemerin concentrations with the presence and severity of diabetic retinopathy in type 2 diabetes mellitus patients. *Archives of Physiology and Biochemistry*.

[21] Du J., Li R., Xu L. (2016). Increased serum chemerin levels in diabetic retinopathy of type 2 diabetic patients. *Current Eye Research*.

[22] Li J., Hu W.-C., Song H., Lin J.-N., Tang X. (2016). Increased vitreous chemerin levels are associated with proliferative diabetic retinopathy. *Ophthalmologica*.

[23] Gu P., Wang W., Yao Y. (2019). Increased circulating chemerin in relation to chronic microvascular complications in patients with type 2 diabetes. *International Journal of Endocrinology*.

